# Curcumin coating: a novel solution to mitigate inherent carbon nanotube toxicity

**DOI:** 10.1007/s10856-024-06789-9

**Published:** 2024-03-25

**Authors:** Samiksha Rele, Chanchal Kiran Thakur, Fatima Khan, Budhadev Baral, Vaishali Saini, Chandrabose Karthikeyan, N. S. Hari Narayana Moorthy, Hem Chandra Jha

**Affiliations:** 1 Infection Bioengineering Group, Department of Biosciences and Biomedical Engineering, Indian Institute of Technology Indore, Simrol, Indore, MP 453552 India; 2 Cancept Therapeutics Laboratory, Department of Pharmacy, Indira Gandhi National Tribal University, Lalpur, Amarkantak, MP 484887 India

## Abstract

**Abstract:**

Multi-walled Carbon Nanotubes (MWCNTs) are inert structures with high aspect ratios that are widely used as vehicles for targeted drug delivery in cancer and many other diseases. They are largely non-toxic in nature however, when cells are exposed to these nanotubes for prolonged durations or at high concentrations, they show certain adverse effects. These include cytotoxicity, inflammation, generation of oxidative stress, and genotoxicity among others. To combat such adverse effects, various moieties can be attached to the surface of these nanotubes. Curcumin is a known anti-inflammatory, antioxidant and cytoprotective compound derived from a medicinal plant called *Curcuma longa*. In this study, we have synthesized and characterized Curcumin coated-lysine functionalized MWCNTs and further evaluated the cytoprotective, anti-inflammatory, antioxidant and antiapoptotic effect of Curcumin coating on the surface of MWCNTs. The results show a significant decrease in the level of inflammatory molecules like IL-6, IL-8, IL-1β, TNFα and NFκB in cells exposed to Curcumin-coated MWCNTs as compared to the uncoated ones at both transcript and protein levels. Further, compared to the uncoated samples, there is a reduction in ROS production and upregulation of antioxidant enzyme-Catalase in the cells treated with Curcumin-coated MWCNTs. Curcumin coating also helped in recovery of mitochondrial membrane potential in the cells exposed to MWCNTs. Lastly, cells exposed to Curcumin-coated MWCNTs showed reduced cell death as compared to the ones exposed to uncoated MWCNTs. Our findings suggest that coating of Curcumin on the surface of MWCNTs reduces its ability to cause inflammation, oxidative stress, and cell death.

**Graphical Abstract:**

(a) Synthesis of Curcumin-coated-Lysine-functionalized MWCNTs. (b) Flow of research depicting experimental groups and studies performed along with the underlying techniques used.
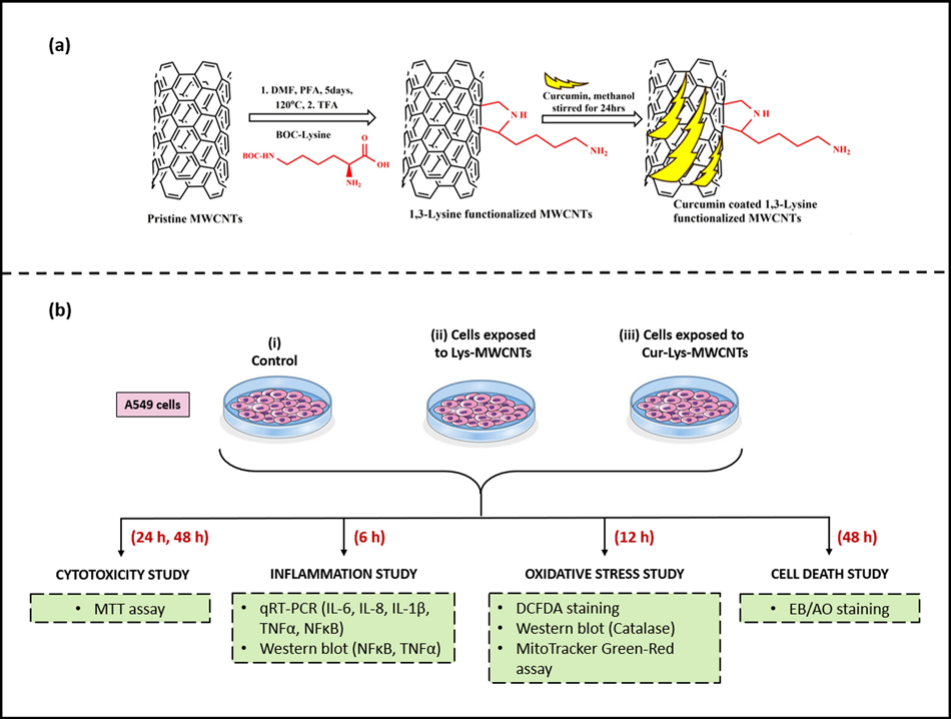

## Introduction

Nanotechnology has the potential to revolutionize many fields, including medicine, electronics, energy, and environmental sciences. At the nanoscale, materials exhibit extraordinary properties that differ significantly from their bulk counterparts [1], making it feasible to design and fabricate new materials with specific properties. Carbon nanotubes (CNTs) are cylindrical structures made of carbon atoms that exhibit unique mechanical, electrical, and thermal properties [2]. Due to their remarkable properties, CNTs have gained attention as promising nanomaterial for use in research and medicine [3, 4]. CNTs display immense potential as vehicles for targeted drug delivery in cancer and many other diseases [5–7]. Studies have shown that drug treatment provided by loading drugs onto such nanocarriers is more effective than direct drug dose due to several different reasons like slower release of drug from the carrier into the tissues and targeted delivery of drugs to cells [8–10]. The high aspect ratio and surface area of CNTs can enhance drug loading and delivery efficiency of therapeutics [11, 12].

However, recent studies have shown that overexposure of living systems to CNTs can lead to potential health hazards like cytotoxicity, inflammation, oxidative stress, genotoxicity, and others [13–16]. CNTs can also accumulate in various organs, including the lungs, liver, spleen, and kidneys, which can lead to organ damage and toxicity [17]. There are also concerns about prolonged exposure to CNTs that may increase the risk of developing cancer [18] or other chronic diseases. Studies have shown that CNTs can induce inflammation and fibrosis in the lungs, which can lead to chronic lung disease [19]. The mechanisms of CNT-induced inflammation involve the activation of immune cells, such as macrophages, and the release of pro-inflammatory cytokines, such as interleukin-1β (IL-1β), interleukin-6 (IL-6) and tumor necrosis factor-alpha [20]. Furthermore, CNTs can also activate the inflammasome, a multiprotein complex that regulates the secretion of IL-1β, resulting in the amplification of the inflammatory response [21].

Oxidative stress is a common mechanism of toxicity associated with CNTs. The generation of reactive oxygen species (ROS) is a major contributor to oxidative stress, and several studies have shown that CNTs can induce ROS production in various cell types and tissues [22]. Additionally, CNTs have been found to penetrate cell membranes and interact with cellular components, which can lead to oxidative stress and inflammation [23]. Studies have also shown that CNTs can induce programmed cell death or apoptosis in different cell lines upon prolonged exposures [24].

Surface modification is an emerging field in nanotechnology which aims towards modifying the surface functional groups of nanomaterials in such a way that it mitigates their adverse effects. Studies have shown that surface modification also helps in increasing the solubility of CNTs [25]. Researchers around the globe are trying to find biocompatible compounds which can be coated onto such nanomaterials to enhance their properties. Curcumin is a suitable candidate molecule for such endeavors as it does not harm the cells and at the same time can potentially mitigate the inflammatory, cytotoxic, and oxidative stress generating activity of MWCNTs. Curcumin, the bioactive ingredient of the medicinal plant *Curcuma longa*, is known for its anti-inflammatory and anti-oxidative properties [26]. Curcumin inhibits NFκB, which is the central mediator of inflammation [27]. Curcumin has been reported to increase the production of antioxidant enzymes and thereby display its antioxidant activity [28]. Its anti-inflammatory activity has been well established on different cell lines, prominently observed in lung carcinoma cell line (A549) [29]. Moreover, the adverse effects of CNTs have been largely reported on pulmonary systems and the results have been established on A549 cell [30, 31]. These two facts made this cell line suitable for the in-vitro experiments in this study.

In this study, we coated lysine-functionalized multi-walled carbon nanotube with Curcumin and performed a comparative study between Curcumin-coated-lysine-functionalized MWCNTs, denoted as Cur, and Lysine-functionalized MWCNTs, denoted as Lys. We evaluated and compared both these compounds at two different concentrations for their cytotoxic, inflammatory, oxidative stress generating, and apoptotic effects on human lung carcinoma cell line (A549). By using Curcumin-coated nanotubes, we showed that these MWCNTs could be potentially used as nanocarriers by efficiently reducing the inflammatory attributes of uncoated CNTs.

## Material and methods

### Material

Pristine multiwalled carbon nanotube (P-MWCNTs), Boc-Lysine was purchased from Sisco Research Laboratories, Mumbai, India. Dimethylformamide (DMF) and Trifluoroacetic acid (TFA) were supplied by M/s Molychem (P) Ltd, India. Paraformaldehyde (PFA) and Curcumin (CU) were procured from M/s Central Drug House (P) Ltd, India. Dialysis membrane: 12 kDa molecular weight cutoff, HiMedia Laboratories, India. For cell culture, Dulbecco’s modified Eagle’s medium (DMEM) and Penicillin-Streptomycin was purchased from Himedia, Mumbai, India and FBS from south American origin; Gibco, USA. TRIzol reagent was purchased from Sigma, USA, TAKARA cDNA synthesis kit from Takara, Japan and SYBR green real-time master mix from Thermo Scientific, USA. Primers for qRT-PCR were procured from IDT, Sweden, and antibodies for western blot from Invitrogen, USA.

### Methods

#### Lysine Functionalized P-MWCNTs by cycloaddition methods

**Fig. 1:**
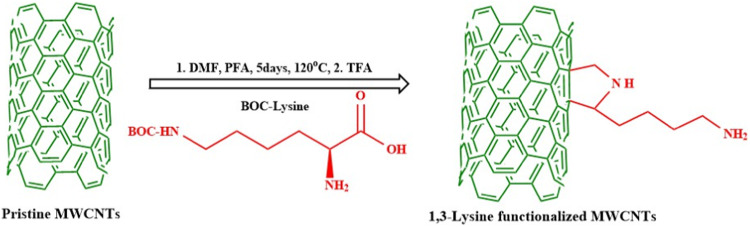
Scheme for 1,3-dipolar cycloaddition functionalization of lysine with pristine MWCNTs

P-MWCNTs (100 mg) was dispersed in 100 ml of DMF and Boc-Lysine and PFA (1:1 molar ratio) were added to this suspension every 24 h for 5 days. The reaction suspension was stirred for 5 days at 130 ^o^C. Upon completion, the unreacted MWCNTs which remained insoluble in DMF was filtered out using Millipore polytetrafluoroethylene filter (0.22 µm pore size) and washed with deionized water. The resulting brown filtrate was evaporated and concentrated under reduced pressure to give a dark brown oily liquid which was collected and then dialyzed (dialysis membrane) in deionized water for 24 h. The retentate was collected and acidified (pH = 4) with hydrochloric acid, then washed once with ethyl acetate and chloroform. Finally, the aqueous layer was collected and basified with sodium hydroxide at 50 ^o^C till it becomes cloudy. The cloudy solution was then allowed to cool to form a layer of brown powder. The powder layer was separated by filtration and washed with water until it became neutral, after which the solid materials were collected and dried under vacuum. The obtained Boc-Lysine functionalized MWCNTs were stirred for 2 h at 50^o^C in TFA to remove the Boc group. The 1,3-lysine functionalized MWCNTs recovered after evaporating the solvent was washed with deionized water and dried using vacuum desiccator [32, 33] (Fig. 1).

#### Curcumin coated 1,3-lysine functionalized MWCNTs

50 mg 1,3-lysine functionalized MWCNTs/ Pristine MWCNTs and 100 mg Curcumin were dissolved in 50 mL methanol and sonicated for 20 min before stirring overnight at room temperature. To eliminate free Curcumin, the Curcumin coated 1,3-lysine functionalized MWCNTs/ Pristine MWCNTs were centrifuged at 10,000 rpm for 15 min [34, 35]. The amount of free Curcumin in the supernatant was determined using UV spectrophotometry and the drug coating efficiency was estimated (DCE). The DCE for Curcumin was determined using the formula given below:-

$$Drug\,coating\,efficency\,( \% )=\frac{wt.\,of\,CU\,added-wt.\,of\,free\,CU}{wt.\,of\,free\,CU\,added}\times 100$$

### Fourier Transform Infrared (FT-IR) spectroscopy

All the products such as P-MWCNTs and 1,3-lysine functionalized MWCNTs were characterized by FT-IR spectroscopy (M/S Bruker, vertex 70, Optic GmbH, Germany) using KBr (potassium bromide) pellet method. The samples (2 mg) were mixed with 100 mg of KBr and triturated properly, subsequently kept into a hydraulic press die, and applied suitable pressure [36]. After this treatment, the collected sample pellets were placed in a sample holder disc of FT-IR spectroscopy for analysis and recorded the spectra at a scanning range of 4000–600 cm^−1^.

### Nuclear Magnetic Resonance (1H NMR) spectroscopy

^1^H NMR spectra of 1,3-lysine functionalized MWCNTs samples were recorded at Panjab University, Chandigarh, India using Avance NEO 500 MHz FT-NMR spectrometer, M/S Bruker, Switzerland. The samples were dissolved in d6-DMSO using a water-bath sonicator and a vortex shaker; the samples were then placed in an NMR sample-tube and the NMR spectra were recorded.

### X-ray diffraction (XRD) analysis

Pristine MWCNTs, 1,3-lysine functionalized MWCNTs, and Curcumin coated 1,3-lysine functionalized MWCNTs were analyzed using a powder XRD Diffractometer (M/S Malvern PANalytical, X Pert3 Powder, ALMELO, Netherlands) and the diffraction values were measured at 2Ɵ values between the range of 10-50 degree.

### Scanning Electron Microscope (SEM) analysis

Scanning electron microscopy (JEOL, JSM7610F Plus) was used to examine the surface morphology of pristine MWCNTs, 1,3-lysine functionalized MWCNTs and Curcumin coated 1,3-lysine functionalized MWCNTs. The developed MWCNTs formulations were dripped into a silicon-based grid and dried at room temperature. The gold-coated MWCNTs formulation was evaluated by SEM for surface morphological characterizations.

### Particle size distribution and Zeta potential studies

Pristine MWCNTs, 1,3-lysine functionalized MWCNTs, and Curcumin coated 1,3-lysine functionalized MWCNTs were dispersed in distilled water and analyzed using a Dynamic Light Scattering instrument (Litesizer 500 from Anton PAAR, Graz, Austrian, and USA) to determine particle size, polydispersity index (PDI), and Zeta potential values.

### Cell culture

Animal cell culture was performed as stated previously [37]. Briefly, human lung carcinoma cell line, A549, was procured from the National Center for Cell Science, Pune, India. The cells were cultured in complete cell culture media containing Dulbecco’s modified Eagle’s medium (DMEM; Himedia, Mumbai, India), 10% fetal bovine serum (FBS south American origin; Gibco, USA) and 100 U/ml penicillin-streptomycin (Himedia, Mumbai, India) under specific conditions of 5% CO_2_ and humidified air at 37 °C (Hera cell 160, Thermo, USA).

### Drug treatment

Lysine functionalized MWCNTs and Curcumin coated lysine functionalized MWCNTs were dissolved in DMSO (5 mg/ml) and sonicated at 50 °C for 15 min in the bath sonicator just prior to treatment. They were subsequently dissolved in cDMEM to achieve a final concentration of 40 μg/ml and 20 μg/ml. Post treatment the cells were incubated for desired time in CO_2_ incubator at 37 °C (Hera cell 160, Thermo, USA).

### Cell survival assay

The MTT assay was performed as previously described [38]. A549 cells were seeded in a 96-well plate at 50% confluency and incubated for 24 h. Uncoated and Curcumin coated MWCNTs were diluted to form 5 concentrations *via* serial dilution namely, 40 μg/ml, 20 μg/ml, 10 μg/ml, 5 μg/ml and 2.5 μg/ml. 0.8% v/v DMSO was used as vehicle control with DMSO concentration equivalent to that in highest MWCNTs concentration i.e., 40 μg/ml. Cells were incubated with these dilutions for 24 and 48 h. Post incubation, media was aspirated and cells were washed with PBS. Then, 50 μL of 0.5 mg/ml MTT dye was added to each well and cells were incubated in dark for 3 h at 37 °C. After incubation, MTT was removed carefully, and formazan crystals were dissolved by the addition 100 μL DMSO to each well. Cells were then incubated for another 2 h in dark on an orbital shaker at 220 rpm. Absorbance was measured using a microplate reader at 570 nm.

### RNA isolation and qRT-PCR

Cells were seeded in a 6-well plate at 50% confluency. At 80% confluency they were treated with Curcumin coated and uncoated MWCNTs at 40 μg/ml and 20 μg/ml concentration. 0.8% DMSO treated cells were kept as vehicle control as the DMSO concentration in 40 μg/ml is 0.8% v/v. After 6 h of incubation, cell pellets were collected. RNA isolation was performed using TRIzol reagent (Sigma, USA) [39]. Two micrograms of RNA was used to prepare cDNA by TAKARA cDNA synthesis kit (Takara, Japan). qRT-PCR was performed using SYBR green real-time master mix (Thermo Scientific, USA) on Agilent AriaMX (Agilent, USA). Gene specific primers obtained from IDT technologies (IDT, Sweden) were used for gene amplification (Table 1).

**Table 1:** List of primers

Sr. No.	Gene	Forward and Reverse primer
1	GAPDH	FP: TGCACCACCAACTGCTTAG RP: GATGCAGGGATGATGTTC
2	IL-6	FP: TACCCCCAGGAGAAGATTCC RP: TTTTCTGCCAGTGCCTCTTT
3	IL-8	FP: GTGCAGTTTTGCCAAGGAGT RP: CTCTGCACCCAGTTTTCCTT
4	IL-1β	FP: GGGCCTCAAGGAAAAGAATC RP: TTCTGCTTGAGAGGTGCTGA
5	NFκB	FP: GAACAGCCTTGCATCTAGCC RP: TCCGAGTCGCTATCAGAGGT
6	TNFα	FP: CAGAGGGCCTGTACCTCATC RP: GGAAGACCCCTCCCAGATAG

### Western blotting

Immunoblotting was performed as mentioned in the pervious manuscript [40]. Cells were seeded in 60 mm tissue culture plates at 50% confluency. Treatment with MWCNTs was given at 80% confluency. Cell pellet was collected after 6 h of incubation for inflammatory proteins and 12 h of incubation for antioxidant enzyme. Protein isolation was performed using RIPA (radioimmunoprecipitation assay) lysis buffer. Protein concentration was estimated using Bradford assay. SDS PAGE was performed by loading an equal amount of total protein in each sample. Separated proteins were transferred onto 0.22 μm nitrocellulose membrane. Membrane blocking was performed using 4.5% BSA. Target proteins were identified by incubation with the following primary antibodies for 2 h in 1:1000 dilution—anti-NF-κB, anti-TNFα, anti-Catalase, and anti-GAPDH. (Invitrogen, USA) After washing, the membrane was incubated for 1 h in horseradish peroxidase-conjugated secondary antibodies against the respective primary antibodies in 1:3000 dilution. Target proteins were detected using chemiluminescence ECL Western blotting substrate (Thermo Scientific, Rockford, IL). Image analysis and quantification was performed using ImageJ software (National Institutes of Health, Bethesda, MA, USA).

### Cellular ROS estimation

Cellular ROS content was measured using DCFDA (2’-7’-Dichlorodihydrofluorescein diacetate) dye as mentioned previously [40]. In short 12 h post incubation with MWCNTs. The live cells were stained with 10 μg/mL of the dye in PBS and incubated for 20–25 min, followed by a wash with PBS, and visualization was done under Olympus IX83 fluorescent microscope (Olympus, Japan) aided with cell Sens imaging software at 20× objective magnification. The amount of intracellular ROS was proportional to DCF fluorescence intensity and was quantified using ImageJ software. Relative changes in DCF fluorescence were expressed as fold increases over the control cells.

### Mito tracker red-green assay

Changes in mitochondria membrane potential were assessed using Mito tracker red-green staining as described [40]. Mito tracker red stains the cellular mitochondria in membrane potential dependent manner, while Mito tracker green binds to the mitochondria independent of their membrane potential and represents the mitochondrial mass. 12 h post exposure to MWCNTs, cells were treated with (200 nM) of Mito tracker red in 500 μL of plain DMEM incubated for 40 min at 37 °C. After incubation cells were washed with PBS followed by treatment of (100 nM) Mito tracker green in 500 μL of serum-free media for 40 min. After incubation cells were washed with PBS and images were taken under Olympus IX83 fluorescent microscope aided with cell Sens imaging software at 20× objective magnification.

### Cell death assay

To measure the number of apoptotic and necrotic cells (NC) in Curcumin-coated and uncoated samples, dual acridine orange (AO): ethidium bromide (EB) (Sigma-Aldrich, St. Louis, MO, USA) staining was carried out [41]. For AO: EB staining cells were seeded at 50% confluency in a 6-well plate. Subsequently, the cells were treated with 40ug/ml and 20ug/ml Curcumin-coated and uncoated MWCNTs. After 48 h of treatment, the cells were stained with 200 μl of dual fluorescent staining solution containing 100 μg/ml of AO and EB each (AO/EB, Sigma, St. Louis, MO). The percentage of the live (L), early (EAC), late apoptotic cells (LAC), and NC was calculated by observing under a fluorescence microscope (FluoView 1000, Olympus America Inc., USA) using 480 and 535 nm excitation filters.

### Statistical analysis

Data were statistically analyzed using a two-tailed Student *t*-test. *P* values were estimated using GraphPad Prism version 8, and *P* values of <0.05, <0.01, and <0.001 were considered statistically significant and represented by *, **, and ***, respectively.

## Results

### Curcumin coated 1,3-lysine functionalized MWCNTs

**Fig. 2:**
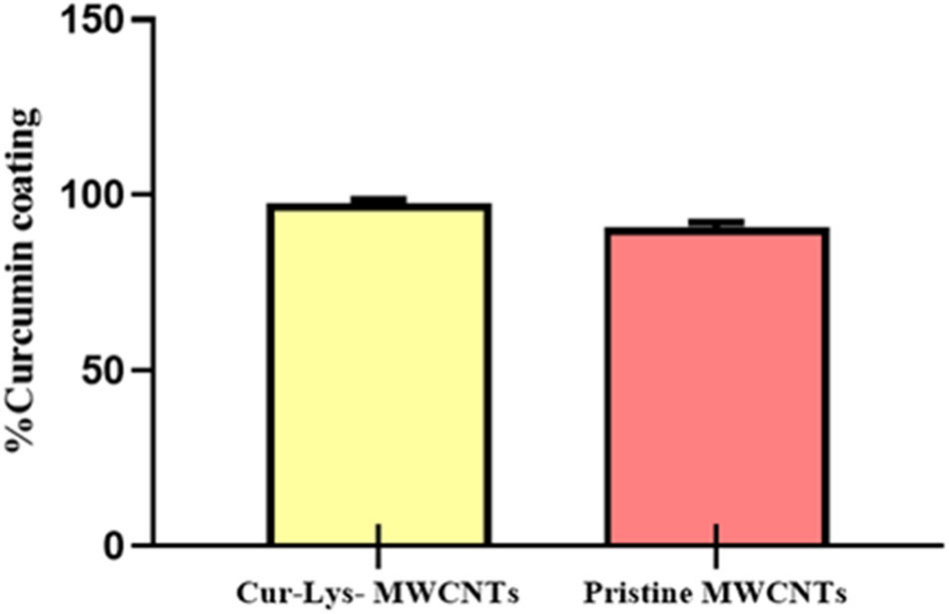
Curcumin coating efficiency

The Curcumin coating efficiency of pristine MWCNTs and 1,3-lysine-functionalized MWCNTs (Cur-Lys-MWCNTs) was determined as 90.72% and 98.77%, respectively (Fig. 2). The results indicate that lysine functionalization of MWCNTs enhanced the Curcumin coating efficiency compared to pristine MWCNTs. This enhancement is attributed to the facilitated surface adsorption of Curcumin on MWCNTs through the π-π interaction of two benzene rings in Curcumin and the formation of hydrogen bonds between the lysine of the functionalized MWCNTs and the phenolic hydroxyl group of Curcumin. Additionally, increased van der Waals interactions between Curcumin and MWCNTs contributed to enhanced Curcumin coating, as both Curcumin and MWCNTs exhibit hydrophobic properties [34, 42, 43].

### Fourier Transform Infrared (FT-IR) Spectroscopy

**Fig. 3:**
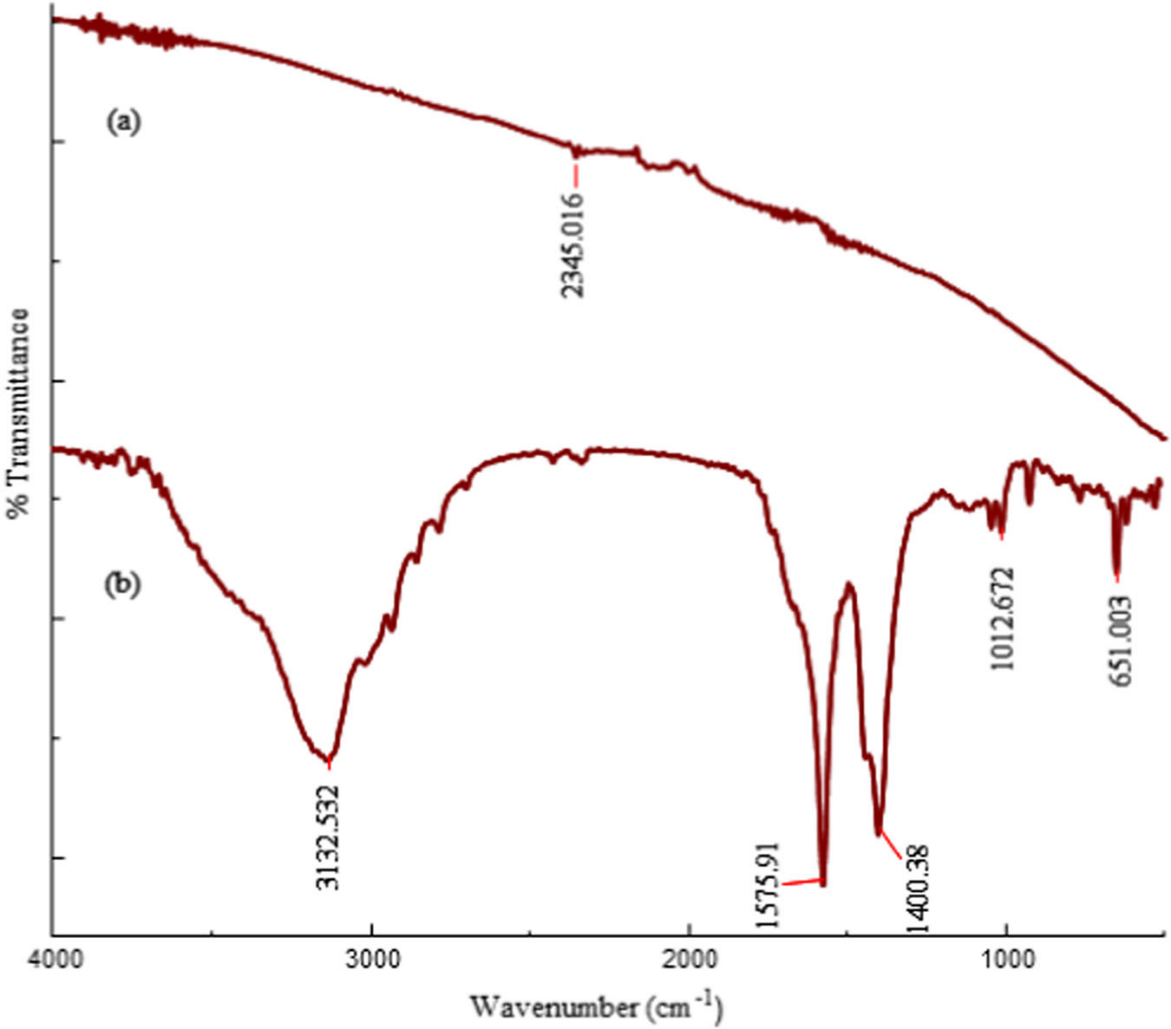
FT-IR spectra of (**a**) pristine MWCNTs and (**b**) 1,3-lysine functionalized MWCNTs

Figure 3 shows the FT-IR spectra of pristine MWCNTs and 1,3-lysine-functionalized MWCNTs. Pure MWCNTs exhibit a peak corresponding to C-H stretching vibration at 2345 cm-1. Pristine MWCNTs exhibit a peak corresponding to C-H stretching vibration at 2345 cm^−1^. FT-IR spectra of 1,3-lysine functionalized MWCNTs, however, shows a peak at 3132 cm^−1^ that is attributable to N-H stretching vibration and the splitting is indicative of a primary amine. Furthermore, the sharp peak at 1571 cm^−1^ is attributable to N-H bending vibration. In addition, the peak at 1400 cm^−1^ corresponds to C-H and C-N vibrations, confirming the successful conjugation of lysine to pure MWCNTs [32, 44].

### Nuclear Magnetic Resonance (^1^H NMR) spectroscopy

**Fig. 4:**
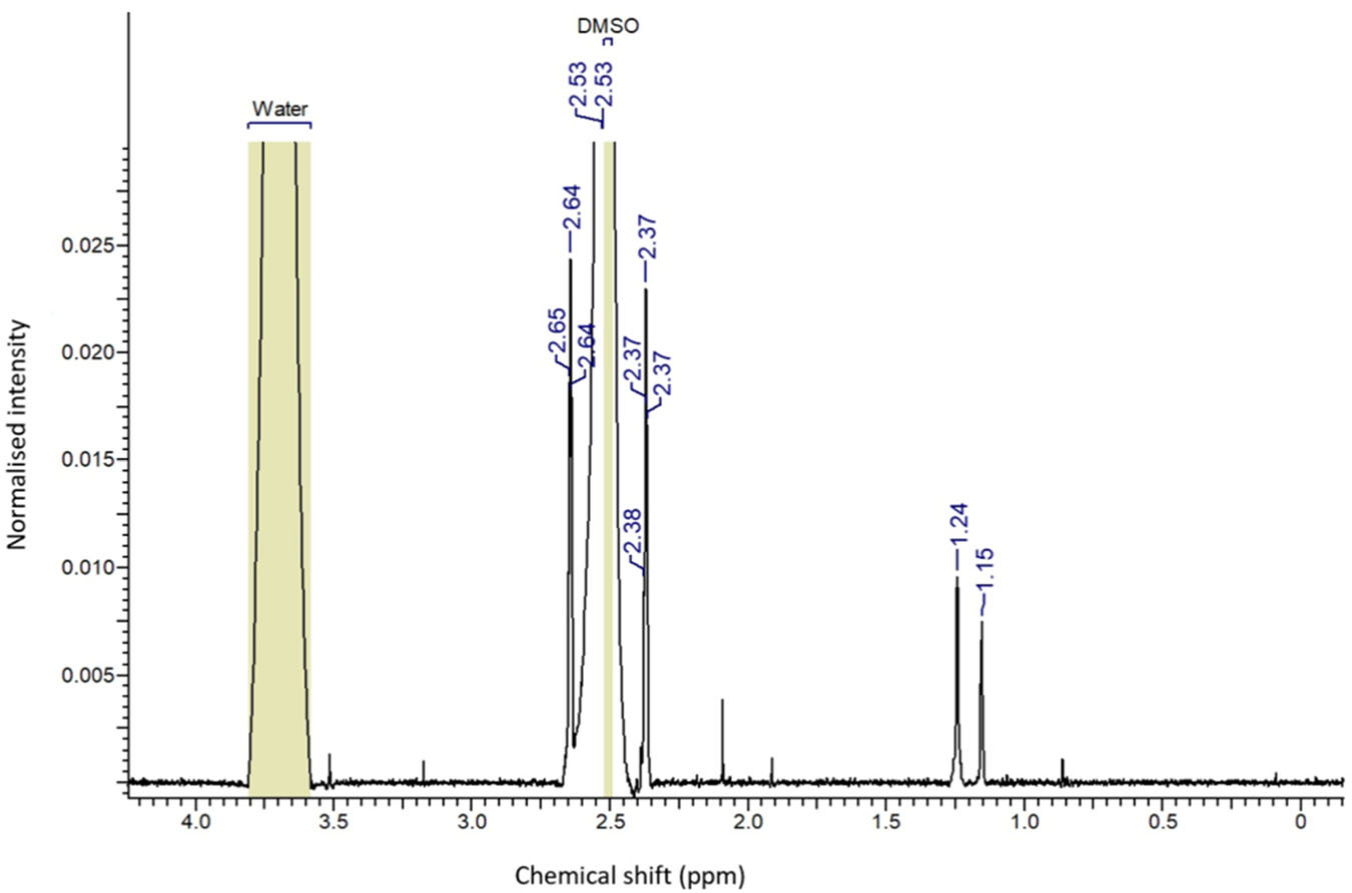
^1^H NMR spectra of 1,3-lysine functionalized MWCNTs

^1^H NMR spectra provided in Fig. 4 confirms the successful synthesis of 1,3-lysine functionalized MWCNTs. The multiple peaks between 1.15 and 2.64 ppm correspond to the methylene protons of lysine, while the peak at 3.51 ppm corresponds to the lysine “NH” proton, indicating that lysine has been effectively conjugated in MWCNTs [32, 45].

### X-ray diffraction (XRD) analysis

**Fig. 5:**
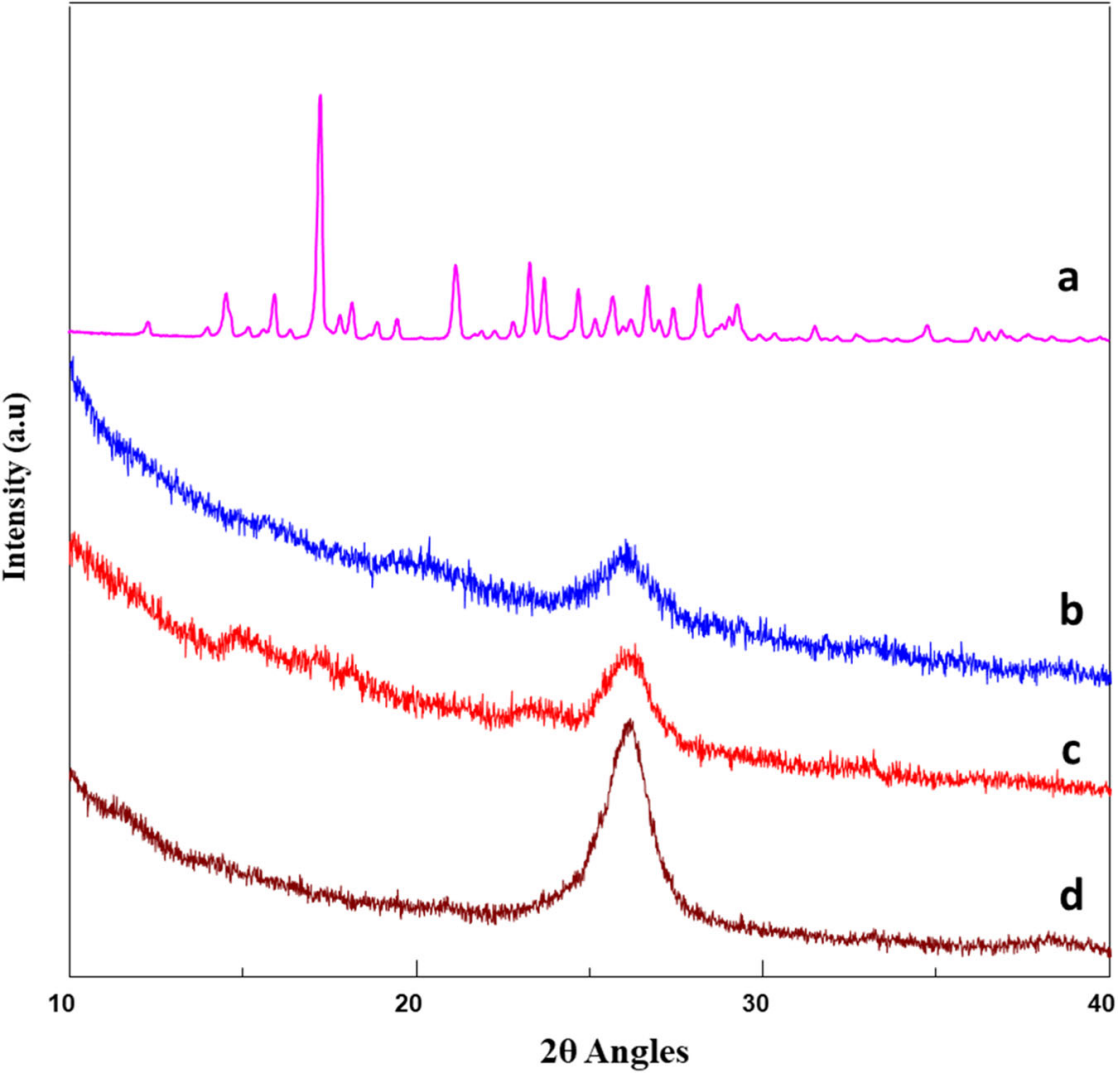
XRD graph of: (**a**) Curcumin, (**b**) Curcumin coated 1,3-lysine functionalized MWCNTs, (**c**) 1,3-lysine functionalized MWCNTs, (**d**) pristine MWCNTs

The XRD graph provides information on the crystallographic structure and chemical composition of the compounds. Figure 5 depicts an XRD graph of pristine MWCNTs and 1,3-lysine- functionalized MWCNTs with similar 2θ angles around 25–30, indicating a very similar amorphous structure. Curcumin exhibited multiple sharp peaks at 2θ angles in the range 10–40, indicating its crystalline nature. The 2θ angles peaks of Curcumin coated 1,3-lysine functionalized MWCNTs exhibited a single broad peak in the range of 25–30, illustrating its amorphous structure and demonstrating that the Curcumin was dispersed on the surface of the MWCNTs [42, 46, 47].

### Scanning Electron Microscope (SEM) analysis

**Fig. 6:**
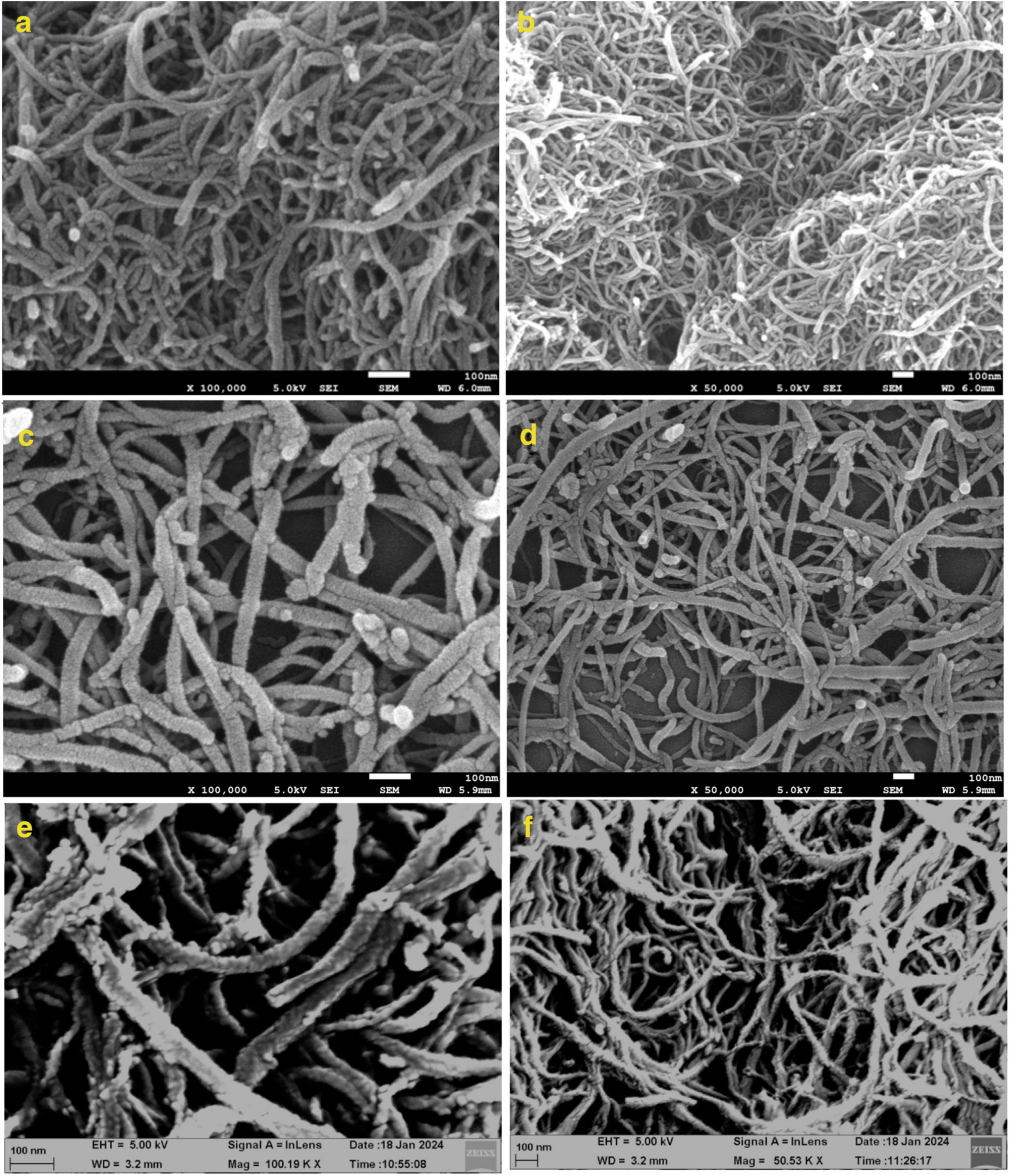
SEM images of: **a**, **b** -1,3-lysine functionalized MWCNTs, **c**, **d** -Curcumin coated 1,3-lysine functionalized MWCNTs and **e**, **f** -Pristine MWCNTs

Figure 6 depicts the SEM analysis of the surface morphology of pristine MWCNTs, 1,3-lysine functionalized MWCNTs, and 1,3-lysine functionalized MWCNTs coated with Curcumin. Their surface morphologies were remarkably similar, indicating that the coating procedure with Curcumin had little effect on the structures of 1,3-lysine functionalized MWCNTs [34, 43].

### Particle size distribution and Zeta potential studies

Table 2 lists the particle size, PDI, and Zeta potential of MWCNT formulations. The results obtained suggest that the increase in size of P-MWCNTs may be indicative of the successful conjugation of lysine and adsorption of Curcumin. The measured PDI values were less than 0.3, indicating the reliability of the system. The measured Zeta potential values of P-MWCNTs indicate that the surface charge is negative (−11.8 mV); however, after lysine conjugation and Curcumin coating, the surface charge became positive (0.6 mV and 0.8 mV, respectively). This may be due to the presence of ε-amino groups that have been protonated. This is advantageous because the presence of a positive charge in nanotubes increases their stability and inhibits aggregation [34, 45].

**Table 2:** Properties of MWCNTs formulations such as particle size, PDI, and Zeta potential

S.no.	Sample	Particle size (*n* = 3)	PDI (*n* = 3)	Zeta analyzer (*n* = 3)
1.	P-MWCNTs	120	0.26	−11.8
2.	1,3-lysine functionalized MWCNTs	161	0.28	0.6
3.	Curcumin coated 1,3-lysine functionalized MWCNTs	230	0.36	0.8

### Curcumin coating on the surface of MWCNTs reduces its cytotoxic effects

**Fig. 7:**
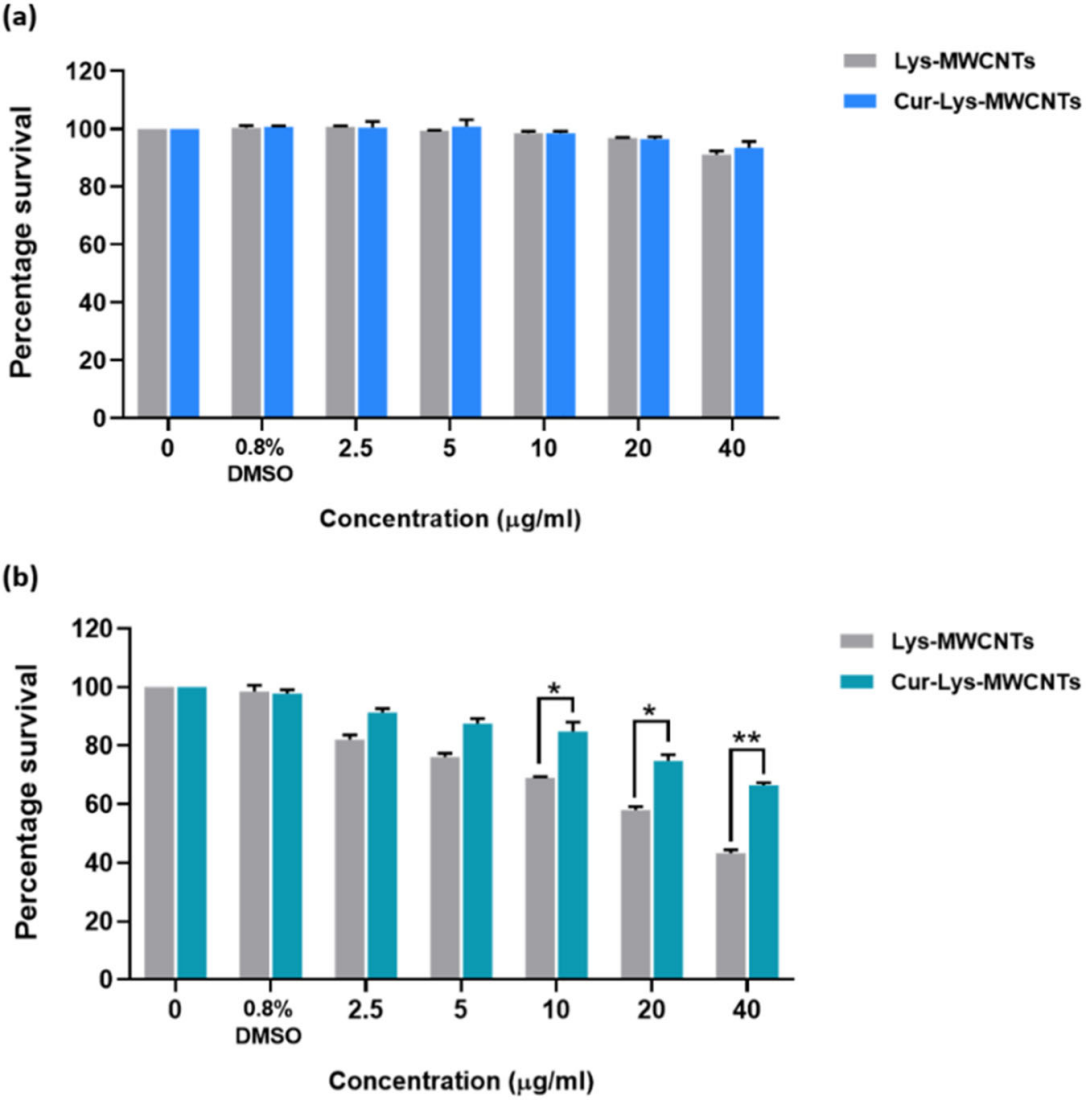
Cell survival percentage graph (**a**) after 24 h incubation with Lys-MWCNTs and Cur-Lys-MWCNTs; **b** after 48 h incubation with Lys-MWCNTs and Cur-Lys-MWCNTs. Here, 0 μg/ml represents media control with no MWCNTs and 0.8% DMSO is vehicle control with DMSO concentration equivalent to that in highest MWCNTs concentration i.e., 40 μg/ml. *P* values of <0.05, <0.01, and <0.001 were considered statistically significant and represented by *, **, and ***, respectively

MTT assay was used to compare the cytotoxic properties of Curcumin-coated and uncoated MWCNTs in lung cell line. After 24 h of incubation (Fig. 7a), both Curcumin coated and uncoated MWCNTs showed no significant cytotoxicity as the survival percentages in both groups remained above 90%. However, after 48 h of incubation, (Fig. 7b) it was observed that the percentage cell survival in the Curcumin-coated MWCNTs (66.4%) was significantly (*p* < 0.01) higher than the uncoated ones (43.2%) at 40 μg/ml concentration.

### Curcumin coating reduced the expression of inflammatory markers after 6 h of exposure to MWCNTs at transcript and protein level

**Fig. 8:**
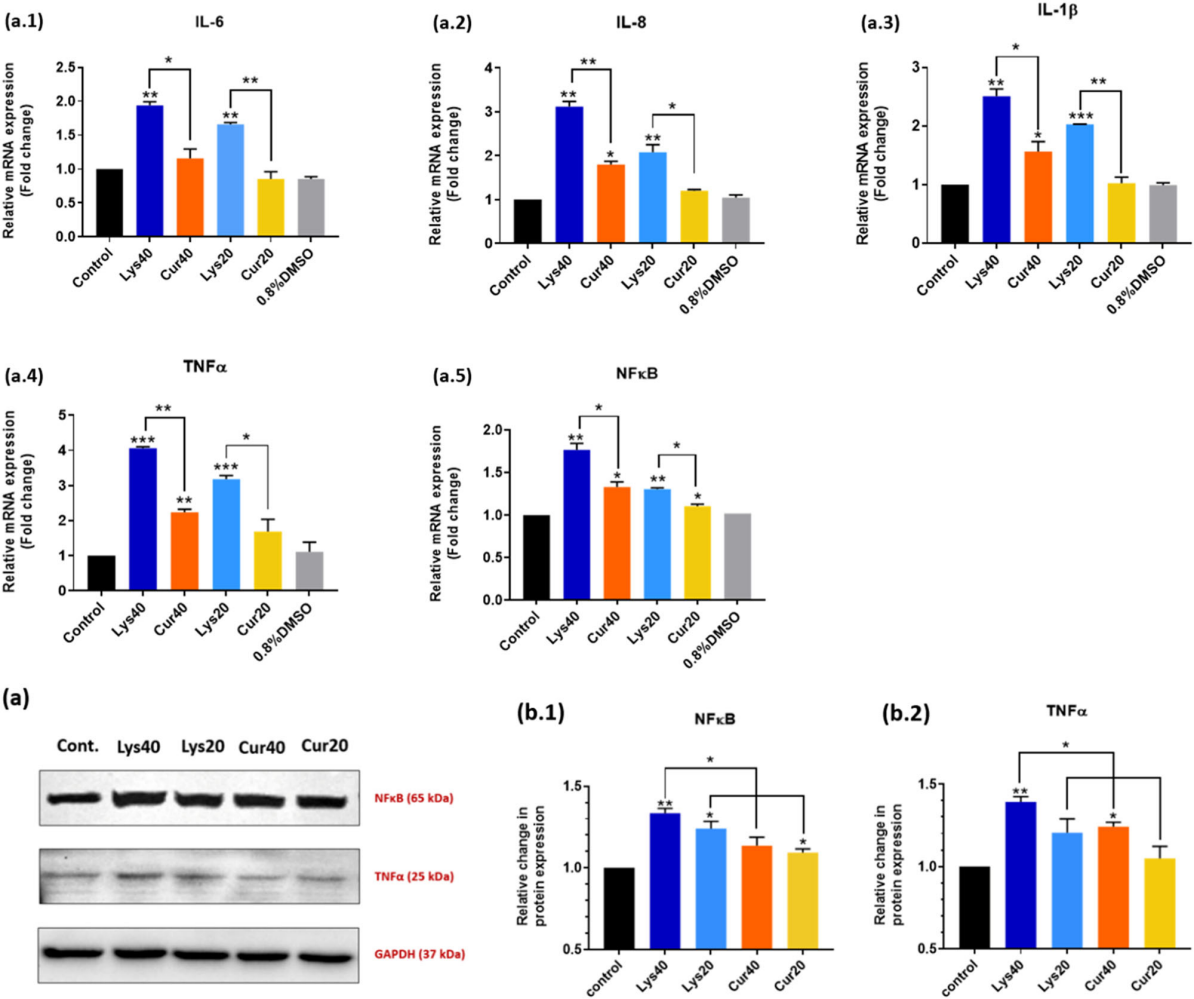
Relative change in mRNA expression (fold change) of inflammatory markers in comparison with GAPDH, (**a**.**1**) IL-6 (**a**.**2**) IL-8 (**a**.**3**) IL-1β (**a**.**4**) TNFα and (**a**.**5**) NFκB after 6 h exposure to Lys-MWCNTs and Cur-MWCNTs at 40 μg/ml and 20 μg/ml concentrations along with media control and DMSO control. **a** Western blot images of protein NFκB, TNFα and GAPDH. Relative change in protein expression of inflammatory markers in comparison with GAPDH, (**b**.**1**) NFκB and (**b**.**2**) TNFα, after 6 h exposure to Lys-MWCNTs and Cur-MWCNTs at 40 μg/ml and 20 μg/ml concentrations along with media control. The results are shown as the mean ± SD from two independent experiments. Asterisk above the bar is in comparison with control and between bars is intergroup unpaired *T*-test. *P* values of <0.05, <0.01, and <0.001 were considered statistically significant and represented by *, **, and ***, respectively

A panel of five inflammatory markers which play a major role in inflammation caused via MWCNTs exposure as previously reported [40] was selected. 6 h of exposure to uncoated MWCNTs at 40 μg/ml significantly (*p* < 0.01) increased the expression of pro-inflammatory cytokine IL-6 while Curcumin-coating significantly (*p* < 0.05) reduced the IL-6 expression at the same concentration (Fig. 8a.1). A similar pattern could be observed at 20 μg/ml concentration as well. Similarly, the expression of the other two pro-inflammatory cytokines IL-8 (Fig. 8a.2) and IL-1β (Fig. 8a.3) also decreased in a familiar pattern at both 40 μg/ml and 20 μg/ml concentrations. NFκB, (Fig. 8a.5) which is the key molecule regulating the inflammatory cascade, and TNFα (Fig. 8a.4) also showed a similar pattern of decrease in expression in the Curcumin-coated samples at both 40 μg/ml and 20 μg/ml concentrations.

The protein expression of NFκB and TNFα was evaluated in cells exposed to Curcumin-coated and uncoated MWCNTs after 6 h of exposure using Western blotting. It was observed that 6 h of exposure to uncoated MWCNTs at 40 μg/ml significantly (*p* < 0.01) increased the expression of NFκB (Fig. 8a and b.1) while Curcumin-coating significantly (*p* < 0.05) reduced NFκB expression at 40 μg/ml. Similarly, TNFα expression (Fig. 8a and b.1) significantly (*p* < 0.01) increased after 6 h of exposure to uncoated MWCNTs at 40 μg/ml while Curcumin coating significantly (*p* < 0.05) decreased its expression.

### Curcumin coating reduced cellular ROS production after 12 h of exposure to MWCNTs

**Fig. 9:**
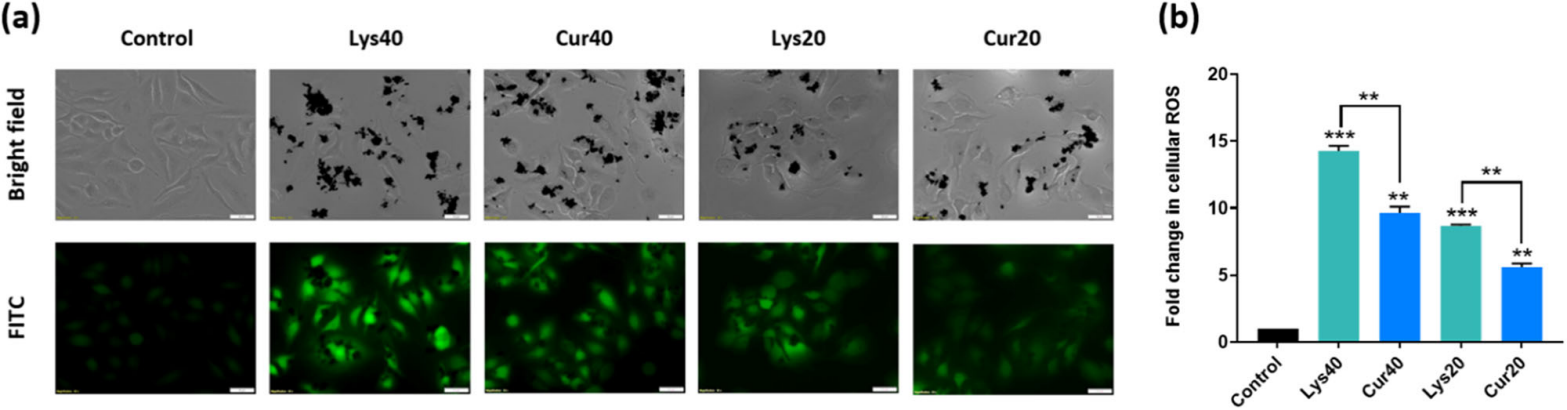
**a** Bright field and FITC filter representative images of live cells after 12 h of exposure to uncoated and Curcumin-coated MWCNTs after performing DCFDA staining. **b** Relative fold change in cellular ROS production w.r.t control after fluorescent intensity quantification using ImageJ software. The results are shown as the mean ± SD from two independent experiments. Asterisk above the bar is in comparison with control and between bars is intergroup unpaired *T*-test. *P* values of <0.05, <0.01, and <0.001 were considered statistically significant and represented by *, **, and ***, respectively

DCFDA staining was performed to check the activity of ROS in cells. Exposure to uncoated MWCNTs at 40 μg/ml concentration for 12 h significantly (*p* < 0.001) increased the ROS activity in the cells as compared to control (Fig. 9b). Curcumin coating led to a significant (*p* < 0.01) reduction in ROS production in cells after 12 h of exposure. Similar pattern could be observed at 20 μg/ml concentration as well.

### Curcumin coating displayed a rescue effect on catalase expression after 12 h of exposure to MWCNTs

**Fig. 10:**
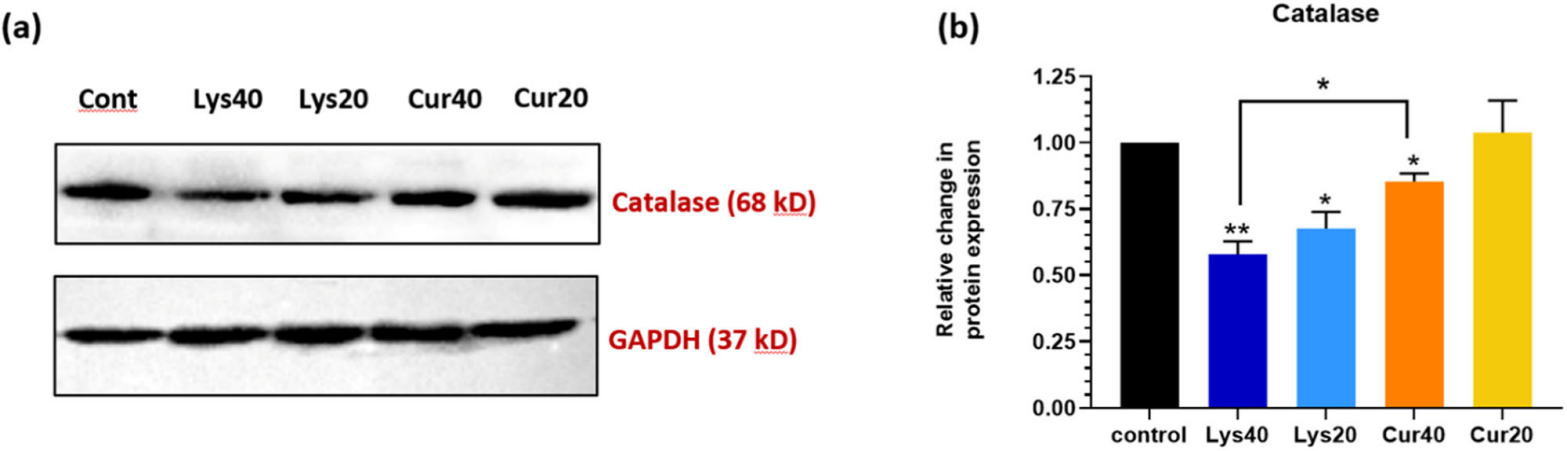
**a** Western blot images of protein Catalase and GAPDH. **b** Relative change in protein expression of antioxidant enzyme-Catalase in comparison with GAPDH after 12 h of exposure to Lys-MWCNTs and Cur-MWCNTs at 40 μg/ml and 20 μg/ml concentrations along with media control. The results are shown as the mean ± SD from two independent experiments. Asterisk above the bar is in comparison with control and between bars is intergroup unpaired *T*-test. *P* values of <0.05, <0.01, and <0.001 were considered statistically significant and represented by *, **, and ***, respectively

Immunoblotting was performed to validate the action of Curcumin coating on the expression of catalase enzyme, wherein after 12 h of incubation of lung cells with MWCNTs it was found that the expression of catalase enzyme had significantly decreased in Lys40 sample (Fig. 10b). Curcumin here showed a rescue effect and significantly increased catalase expression. A similar effect was observed in the 20 μg/ml concentration. The fold changes were measured with respect to GAPDH which is constitutively expressed in all cells.

### Curcumin coating prevented the loss of mitochondrial membrane potential caused due to exposure of MWCNTs for 12 h

**Fig. 11:**
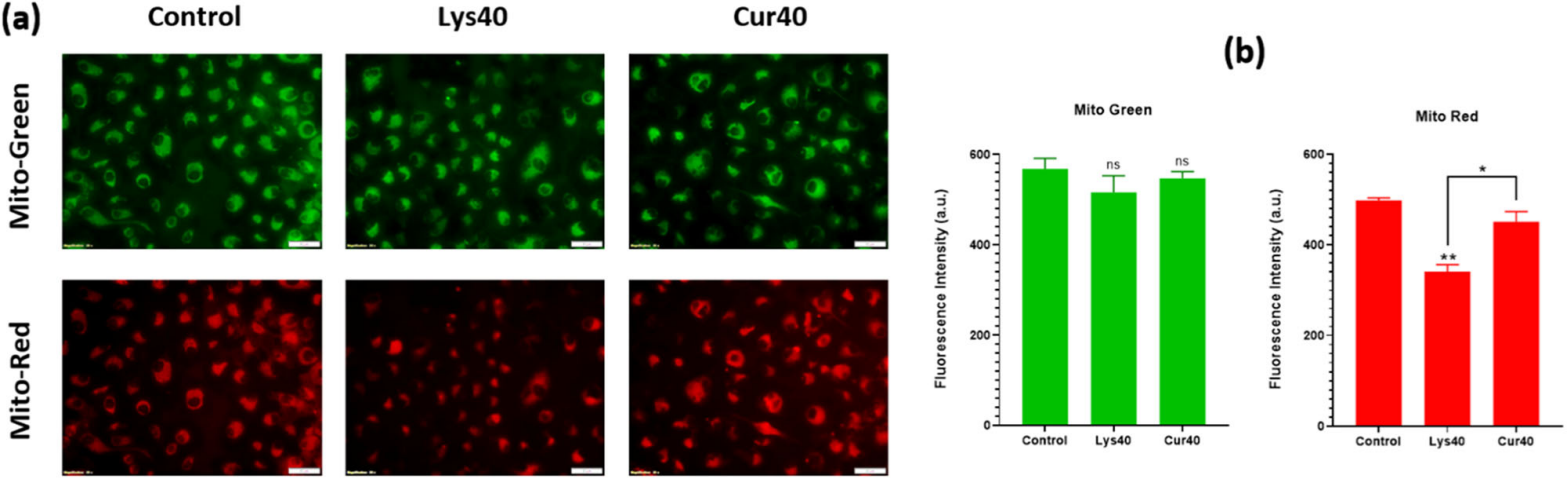
**a** Mito-Green and Mito-Red representative images of live cells after 12 h of exposure to uncoated and Curcumin-coated MWCNTs after performing Mito tracker Green-Red staining assay. **b** Quantified Mito-Green and Mito-Red fluorescent intensities using ImageJ software. The results are shown as the mean ± SD from two independent experiments. Asterisk above the bar is in comparison with control and between bars is intergroup unpaired *T*-test. *P* values of <0.05, <0.01, and <0.001 were considered statistically significant and represented by *, **, and ***, respectively

Through Mito tracker Green-Red staining it was observed (Fig. 11b) that all three groups- Control, Lys40 and Cur40 had nearly constant green fluorescent signal intensity indicating that there was no change in the number of mitochondria in the cell after MWCNTs exposure. However, it could be observed (Fig. 11b) that exposure to uncoated MWCNTs at 40 μg/ml concentration for 12 h significantly (*p* < 0.01) lowered Mito-red fluorescent intensity. Curcumin coating on MWCNTs significantly (*p* < 0.05) recovered the mitochondrial membrane potential as can be seen by an increase in Mito-red fluorescent intensity as compared to uncoated samples.

### Curcumin coating on MWCNTs reduces cell death caused due to their exposure

**Fig. 12:**
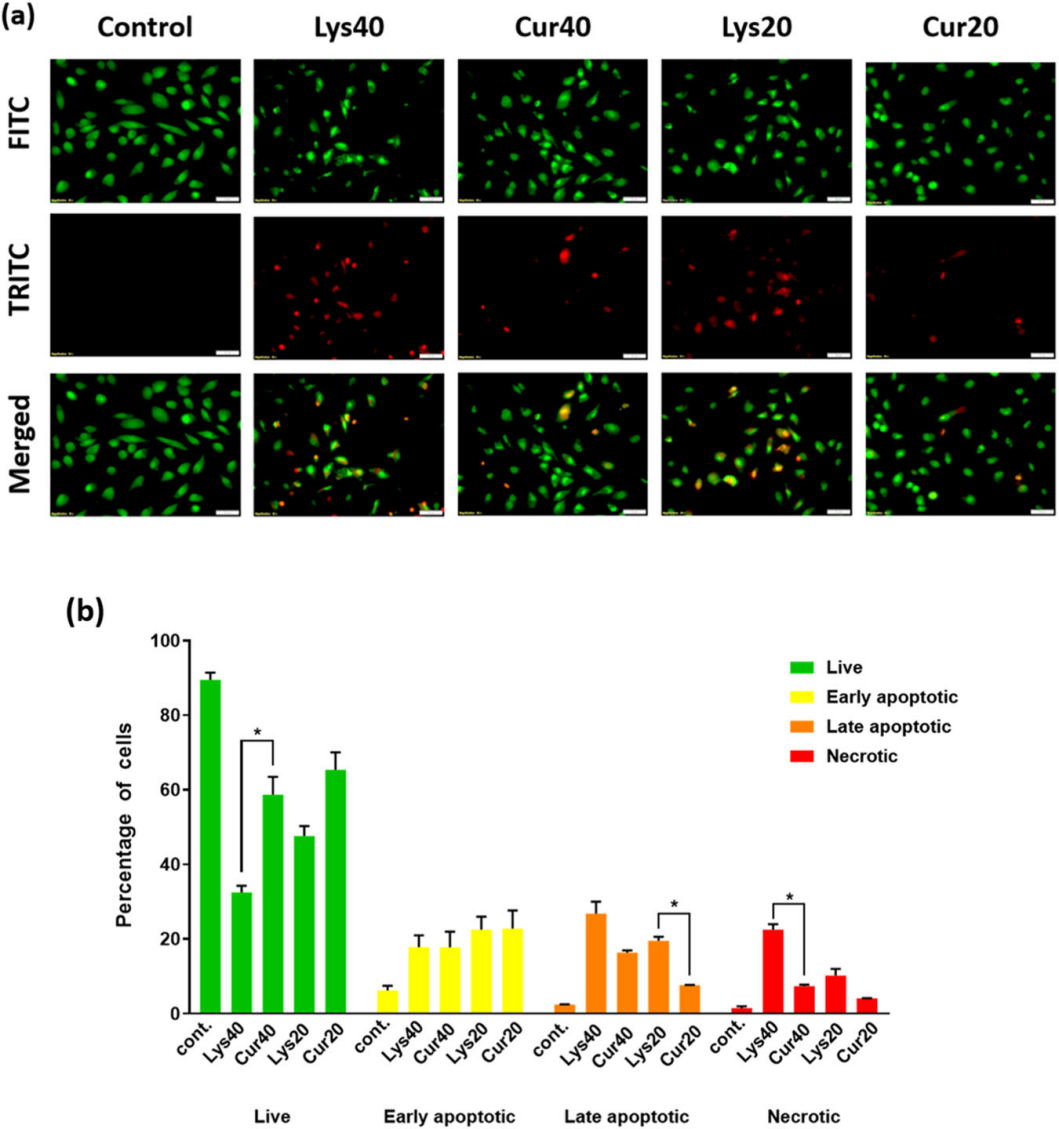
**a** EB/AO staining representative images of cells after 48 h of exposure to uncoated and Curcumin-coated MWCNTs. **b** Percentage of cells under each category: Live (green), Early apoptotic (yellow), Late apoptotic (orange) and Necrotic (red), calculated by counting minimum 200 cells per sample. The results are shown as the mean ± SD from two independent experiments. *P* values of <0.05, <0.01, and <0.001 were considered statistically significant and represented by *, **, and ***, respectively

EB and AO dual fluorescent staining method was performed to check if Curcumin coating was able to delay apoptosis in lung cells after exposure for 48 h. It could be observed (Fig. 12b) that the percentage of live cells was significantly (*p* < 0.05) lower in the Lys40 (32%) and Lys20 (47%) sample groups in comparison to Cur40 (60%) and Cur20 (65%) samples. The percentage of LACs was higher in uncoated MWCNT groups and lower in Curcumin coated groups. The percentage of necrotic cells was highest in the Lys40 group (22.5%) and it was significantly lower (*p* < 0.05) in the Cur40 group (7.4%).

## Discussion

Nanotechnology harnesses the unique properties of materials at nanoscale to create innovative solutions across diverse fields, especially biomedical sciences. CNTs play a crucial role in the field of nanotechnology. They have numerous and diverse applications in the field of biomedical engineering [48]. They are majorly being explored for creating new drug delivery systems for cancer and many other diseases [49]. They have depicted great promise in the field of targeted drug delivery as well [50].

However, in recent years, many reports have shown the adverse effects of exposure to nanotubes on cells and living systems [51]. According to reports, MWCNTs have a number of negative impacts on cells, including cytotoxicity, inflammation, oxidative stress, etc. [15, 52]. Surface modification technology is an emerging field in nanotechnology which aims to modify the surfaces of nanomaterial with attachment or coating of suitable compounds such that the negative effects are minimized enhancing the desired properties. In such scenarios, coating nanotubes with biocompatible natural compounds with beneficial properties is a promising alternative. In our study, we utilized Curcumin coated MWCNTs thereby attributing anti-inflammatory and antioxidant properties to these nanotubes. The aim of this study was to test if Curcumin coating was successful in mitigating the toxic effects of MWCNTs in vitro.

This study validated that Curcumin coated MWCNTs indeed displayed lesser cytotoxicity as compared to the uncoated MWCNTs. This is in alignment with the previous reports which have proved the cytoprotective nature of several plant-derived compounds including Curcumin on cells [53, 54]. In this study, we observed that a 6 h exposure to MWCNTs led to a significant increase the expression of inflammatory proteins and pro-inflammatory cytokines in the cells. This was found to be in accordance with the previous reports proving induction of inflammation upon CNT exposure on various cell lines [55]. However, Curcumin coating led to a significant decrease in the expression of inflammatory markers at both transcript and protein levels. The possible mechanism for this may be inhibition of NFκB, the central mediator for inflammation and transcription factor for various cytokines and chemokines by Curcumin. NFκB inhibition further leads to suppression of pro-inflammatory cytokines like IL-6, IL-8, IL-1β, and TNFα which leads to an overall decrease in inflammation in the cells [56].

The other mechanism via which CNTs exert their toxicity is through production of ROS in the cells. Our study validated that after 12 h exposure to MWCNTs, there was a significant increase in cellular ROS production. This might be due to suppression in production of antioxidant enzymes in the cells by CNT interaction. A study reported downregulation of Catalase expression upon SWCNT exposure on A549 cell line [57]. The molecular mechanisms behind the same remain unknown. Curcumin coating showed a recovery effect that increased the expression of Catalase enzyme in the cells as compared to the cells treated with uncoated MWCNTs. This might be the reason behind reduced ROS activity in the cells exposed to Cur-MWCNTs.

In this study, we also explored the involvement of mitochondria in MWCNT mediated toxicity and found that a 12 h exposure to uncoated MWCNTs caused a drop in the mitochondrial membrane potential. This might be an indicator of mitochondrial damage which can contribute to ROS imbalance in the cells [58]. On the other hand, Curcumin coated MWCNTs led to a lesser drop of MMP indicating that Curcumin is preventing mitochondrial damage. This might be another reason behind reduced oxidative stress on cells as mitochondria is the major cell organelle responsible for maintenance of cellular oxidative homeostasis [59]. Lastly, Curcumin coating on MWCNTs led to reduced cell death as could be observed from the results of cell death assay. Curcumin efficiently delayed apoptosis in cells as there were lesser percentages of LACs in Curcumin coated samples. It significantly reduced necrosis as well. There might be several reasons for this cytoprotective effect of Curcumin on the cells, reduced inflammation, and oxidative stress can be few such reasons as they are the major drivers towards cell death [60, 61].

From existing sources of literature and research we can gauge the huge impact that usage of CNTs can have in the field of medical sciences. However, its potential gets limited due to the adverse effects that it can have on the living systems if exposed to high doses or prolonged durations. This study is first one of its kind where we have tried to mitigate the adverse effects of such nanostructures by coating it with a biochemical agent that combats them. Further, pre-clinical studies need to be performed to validate our in vitro findings.

One of the challenges that were faced while conducting this study was the limited dispersibility of CNTs. This was overcome by two measures: one was by using lysine-functionalized MWCNTs as they have greater dispersibility than the pristine/non-functionalized ones in aqueous solutions [32, 62]; and other was by sonicating the nanotubes in a bath sonicator for 15 min prior to treatment. This helped to significantly improve their dispersibility. Still, minor amount of nanotube aggregations was observed during the study which could have possibly influenced the effects that we observed upon treatment. Yet another challenge lies in sustained attachment of Curcumin molecules to MWCNT surface throughout the experiment. It is difficult to validate if the Curcumin molecules that exhibit the protective effect are the ones that remain attached to the MWCNT surface or the ones that are released. Either way we can assertively conclude that Curcumin coating does help in mitigating the adverse effects. However, we cannot comment on the physical state of Curcumin from this study. Additionally, not all drugs will be compatible to be loaded into a Curcumin-coated CNT, as some might inherently interact with the biomolecule- Curcumin and render the drug to be ineffective.

**Fig. 13:**
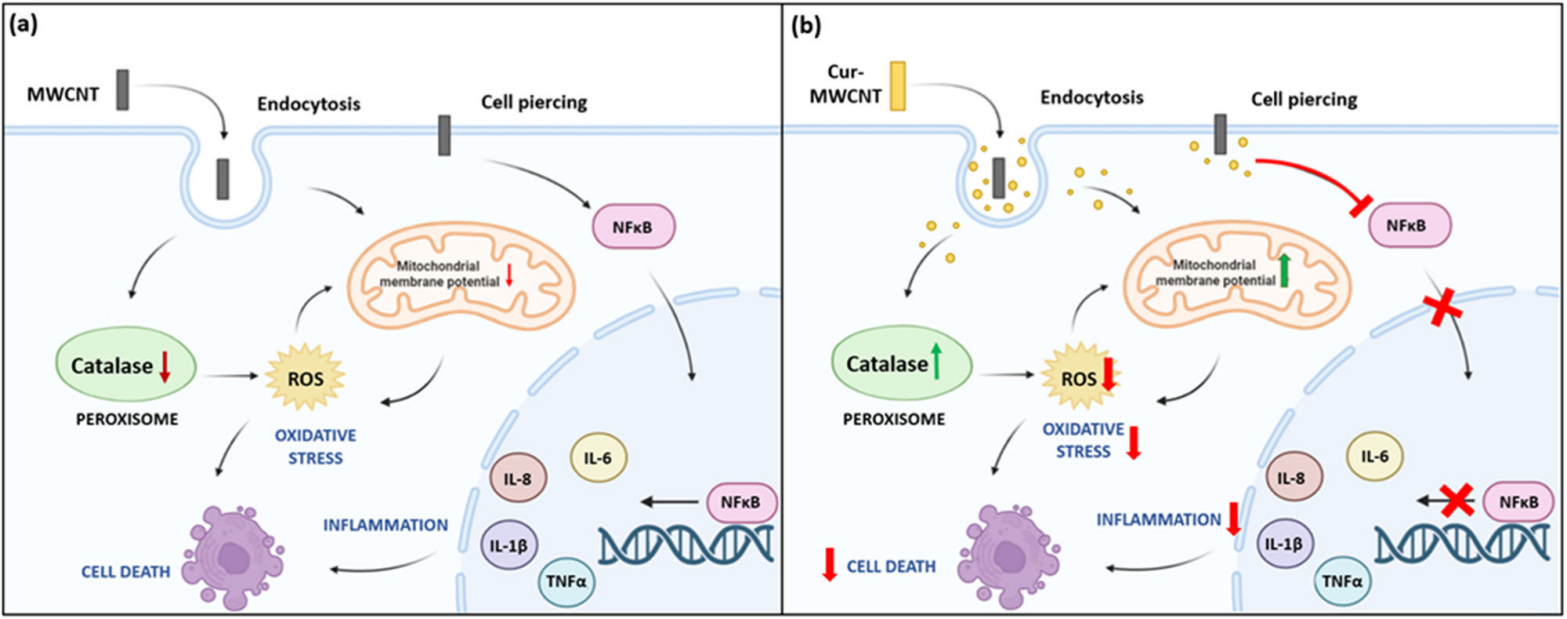
Mechanism of action of (**a**) uncoated MWCNTs and (**b**) Curcumin-coated MWCNTs on cell

A few alternative explanations for the results that we observed in the study can be that Curcumin molecules might be masking or neutralizing the specific components of the carbon nanotube that are responsible for causing toxicity. Another confounding factor here can also be that the coating process itself might induce some minor structural changes in the nanotubes leading to reduced toxicity. There might be a potential synergistic effect of Curcumin and MWCNTs that enhance cellular protection rather than individual ones like we discussed. There can be other molecular pathways as well which can be triggered leading to the effects, we observed apart from the ones we have explored in this study. We believe that this study will inspire researchers in this field to explore different biomaterials for coating of CNTs and to explore the different molecular pathways involved therein. This can help in expanding the usage CNTs in the domain of biotechnology (Fig. 13).

In conclusion, this study indicates that Curcumin coating on the surface of multi-walled CNTs mitigates its cytotoxic, inflammatory, oxidative stress generating, and apoptotic/necrotic effects.

## Data Availability

All the data is included in the main manuscript and supplementary Information.
